# Multiparametric ultrasomics of significant liver fibrosis: A machine learning-based analysis

**DOI:** 10.1007/s00330-018-5680-z

**Published:** 2018-09-03

**Authors:** Wei Li, Yang Huang, Bo-Wen Zhuang, Guang-Jian Liu, Hang-Tong Hu, Xin Li, Jin-Yu Liang, Zhu Wang, Xiao-Wen Huang, Chu-Qing Zhang, Si-Min Ruan, Xiao-Yan Xie, Ming Kuang, Ming-De Lu, Li-Da Chen, Wei Wang

**Affiliations:** 1 Department of Medical Ultrasonics, Institute of Diagnostic and Interventional Ultrasound, The First Affiliated Hospital of Sun Yat-Sen University, 58 Zhongshan Road 2, Guangzhou, 510080 People’s Republic of China; 2 Department of Medical Ultrasonics, The Sixth Affiliated Hospital of Sun Yat-sen University (Guangdong Gastrointestinal Hospital), Guangzhou, China; 3 Research Center of GE Healthcare, Shanghai, China; 4 Zhongshan School of Medicine, Sun Yat-sen University, Guangzhou, China; 5 Department of Hepatobiliary Surgery, The First Affiliated Hospital of Sun Yat-Sen University, Guangzhou, China

**Keywords:** Ultrasonography, Liver fibrosis, Machine learning, Decision support techniques, Data mining

## Abstract

**Objective:**

To assess significant liver fibrosis by multiparametric ultrasomics data using machine learning.

**Materials and Methods:**

This prospective study consisted of 144 patients with chronic hepatitis B. Ultrasomics—high-throughput quantitative data from ultrasound imaging of liver fibrosis—were generated using conventional radiomics, original radiofrequency (ORF) and contrast-enhanced micro-flow (CEMF) features. Three categories of features were explored using pairwise correlation and hierarchical clustering. Features were selected using diagnostic tests for fibrosis, activity and steatosis stage, with the histopathological results as the reference. The fibrosis staging performance of ultrasomics models with combinations of the selected features was evaluated with machine-learning algorithms by calculating the area under the receiver-operator characteristic curve (AUC).

**Results:**

ORF and CEMF features had better predictive power than conventional radiomics for liver fibrosis stage (both *p* < 0.01). CEMF features exhibited the highest diagnostic value for activity stage (both *p* < 0.05), and ORF had the best diagnostic value for steatosis stage (both *p* < 0.01). The machine-learning classifiers of adaptive boosting, random forest and support vector machine were found to be optimal algorithms with better (all mean AUCs = 0.85) and more stable performance (coefficient of variation = 0.01–0.02) for fibrosis staging than decision tree, logistic regression and neural network (mean AUC = 0.61–0.72, CV = 0.07–0.08). The multiparametric ultrasomics model achieved much better performance (mean AUC values of 0.78–0.85) than the features from a single modality in discriminating significant fibrosis (≥ F2).

**Conclusion:**

Machine-learning-based analysis of multiparametric ultrasomics can help improve the discrimination of significant fibrosis compared with mono or dual modalities.

**Key Points:**

*• Multiparametric ultrasomics has achieved much better performance in the discrimination of significant fibrosis (≥ F2) than the single modality of conventional radiomics, original radiofrequency and contrast-enhanced micro-flow.*

*• Adaptive boosting, random forest and support vector machine are the optimal algorithms for machine learning.*

**Electronic supplementary material:**

The online version of this article (10.1007/s00330-018-5680-z) contains supplementary material, which is available to authorized users.

## Introduction

The early detection and accurate staging of liver significant fibrosis are crucial for antiviral therapy. Shear wave elastography (SWE), an elasticity-based US technique, has shown good accuracy in detecting fibrosis [1]. However, the applicability of SWE is substantially limited in cases of obesity, ascites or necroinflammatory activity (up to 15.8%) [2, 3]. Thus, assessment with a single imaging modality only provides limited information and could always be affected by steatosis and necroinflammatory activity [4, 5].

Radiomics, a term that includes the suffix “-omics,” generates high-throughput data from medical images [6, 7], which contain information on prognosis, response to treatment and monitoring of disease status [8, 9]. As one important modality of medical imaging, US can provide not only morphological information but also stiffness and perfusion assessments, which may not be acquired using other imaging methods [5, 10–12]. We have applied the “-omics” concept to computing quantitative US imaging, a field referred to as “ultrasomics.” In our opinion, big imaging data of liver fibrosis, in terms of heterogeneity, tissue texture, stiffness and vascularity perfusion, should be taken into consideration when analyzing fibrosis staging.

In addition to multimodality data, machine learning is another powerful tool to improve clinical decision-making [13]. Currently, newer advances in data analysis contributed by the field of machine learning have greatly extended researchers’ ability to make meaningful discoveries. Machine learning enables accurate and reliable prediction using data with very large numbers of variables and small sample sizes. Therefore, the optimal machine-learning model for ultrasomics studies with small sample sizes should be determined. To our knowledge, comparative studies on the effectiveness of machine learning-based decision support systems are lacking [14].

In this study, we present the concept of multiparametric ultrasomics, which is a machine learning-based clinical decision support system that uses US imaging big data. We extracted a set of ultrasomic features that captures the morphology and hemodynamic changes associated with liver fibrosis to (1) develop a robust, noninvasive technique to predict the liver fibrosis stage using routine US data that can be easily obtained in the clinical setting and (2) investigate the optimal machine-learning model in a small sample size study.

## Materials and methods

### Study population

This prospective study was approved by the Institutional Review Board of our hospital, and informed consent was obtained. From October 2013 to April 2015, a total of 144 hepatitis B virus (HBV) patients who met the eligibility criteria were included in the study. The inclusion and exclusion criteria are detailed in the Supplementary Materials. For each patient, comprehensive blood tests (aspartate transaminase (AST), alanine transaminase (ALT), serum albumin, g-glutamyltransferase, total bilirubin, and platelet count) were performed no more than 3 days before the surgery or biopsy. Combinations of simple markers such as the aspartate aminotransferase-to-platelet ratio index (APRI) and fibrosis-4 index (FIB-4) were calculated; the formula is provided in the Supplementary Materials [15].

### Liver histology analysis

All patients included in the study underwent partial liver surgery (*n* = 61) or biopsy (*n* = 83). Resected liver specimens approximately 10 mm × 10 mm in size were preserved intraoperatively. A US-guided percutaneous liver biopsy of the right lobe was performed with an 18-gauge needle (Bard) within 3 days after ultrasonography. All specimens were fixed in formalin, embedded in paraffin and stained with hematoxylin-eosin (H&E) and Masson. Two liver pathologists with > 10 years of experience, who were blinded to the results of imaging but not to the clinical and biochemical data of the patient, analyzed the specimens. Liver fibrosis was evaluated according to the METAVIR scoring system as follows: F0, no fibrosis; F1, portal fibrosis without septa; F2, portal fibrosis and few septa; F3, numerous septa without cirrhosis; F4, cirrhosis. Significant fibrosis was defined as a score of F2 or greater. The METAVIR system was used to score the intensity of necroinflammatory activity (mainly necrosis) as follows: A0 = no necroinflammatory activity, A1 = mild activity, A2 = moderate activity and A3 = severe activity [4]. Steatosis was scored, using a four-grade scoring system, from S0 to S4: S0 = no steatosis; S1 = mild (1–5%) (% of hepatocytes containing visible macrovesicular steatosis); S2 = moderate (6–32%); S3 = marked (33–66%); S4 = severe (67–100%) [4, 16, 17].

### Multiparametric ultrasomics acquisition and feature extraction

All US examinations were performed with the Aplio 500 scanner (Canon Medical System) equipped with a 375BT convex transducer (frequency, 3.5 MHz). US examinations were performed by one of two radiologists (X.Y.X. and W.W.) with at least 10 years of experience with routine US. Three types of parameters were acquired:

1. B-mode images in digital imaging and communications in medicine (DICOM) format. Images were obtained with intercostal oblique scanning and were expected to show the liver parenchyma from the right intercostal space to the segment 6 region of the right hepatic lobe. Display depth and transmit focus were fixed at 6 cm and 4 cm, respectively, with the receive gain equal to 80. Large vessels defined as > 2.0 mm were avoided. The settings, including time-gain compensation, dynamic range, focal length and mechanical index, were optimized for each examination. Conventional images in DICOM format were stored on the Canon Medical System platform. DICOM images were used to extract conventional radiomics features using A. K. software (Ultrasomics Kit, version 1.0, ZhiXing-Tech), including the first-order intensity statistics, texture and wavelet features. Mathematical definitions of all radiomic features were previously described and are detailed in the Supplementary Materials [18].
2. Radiofrequency-based raw data. The same scanning method and planes of conventional radiomics were used, but data were stored as raw data. Post-beam-formed original radiofrequency data (ORF features) with intact frequency information were used to extract the statistical features of the acquired echo amplitude of the raw data.
3. Dynamic contrast-enhanced micro-flow (CEMF) images. Contrast harmonic imaging was used with a mechanical index of 0.08. The transmission frequency used in contrast harmonic imaging was 3.5 MHz and a frame rate of 15–18 frames per second. Images were obtained with intercostal oblique scanning and were expected to show the liver parenchyma from the right intercostal space to the segment 6 region of the right hepatic lobe and the right kidney on a single screen. Focus was set at a depth of 6 to 8 cm to visualize the kidney. After the contrast harmonic imaging mode was activated, a bolus injection of 2.4 ml of SonoVue (Bracco) was administered intravenously via an antecubital vein, followed immediately by a 5 ml saline flush. Patients were instructed to hold their breath after the injection for 7–8 s (1–2 s before the visualization of renal artery), and then the CEMF mode was initiated. Image acquisition proceeded until the liver was wholly enhanced. Clips obtained for approximately 15–20 s immediately after SonoVue infusion were saved as clip data in DICOM format. Dynamic CEMF features were extracted via our built-in model through off-line analysis. The model was developed with the understanding that the liver received dual blood supply from the hepatic artery and portal vein. The blood supply of the kidney was set as the “hepatic artery supply” and was used as a comparison indicator to reduce the influence of circulation difference.

**Fig. 1:**
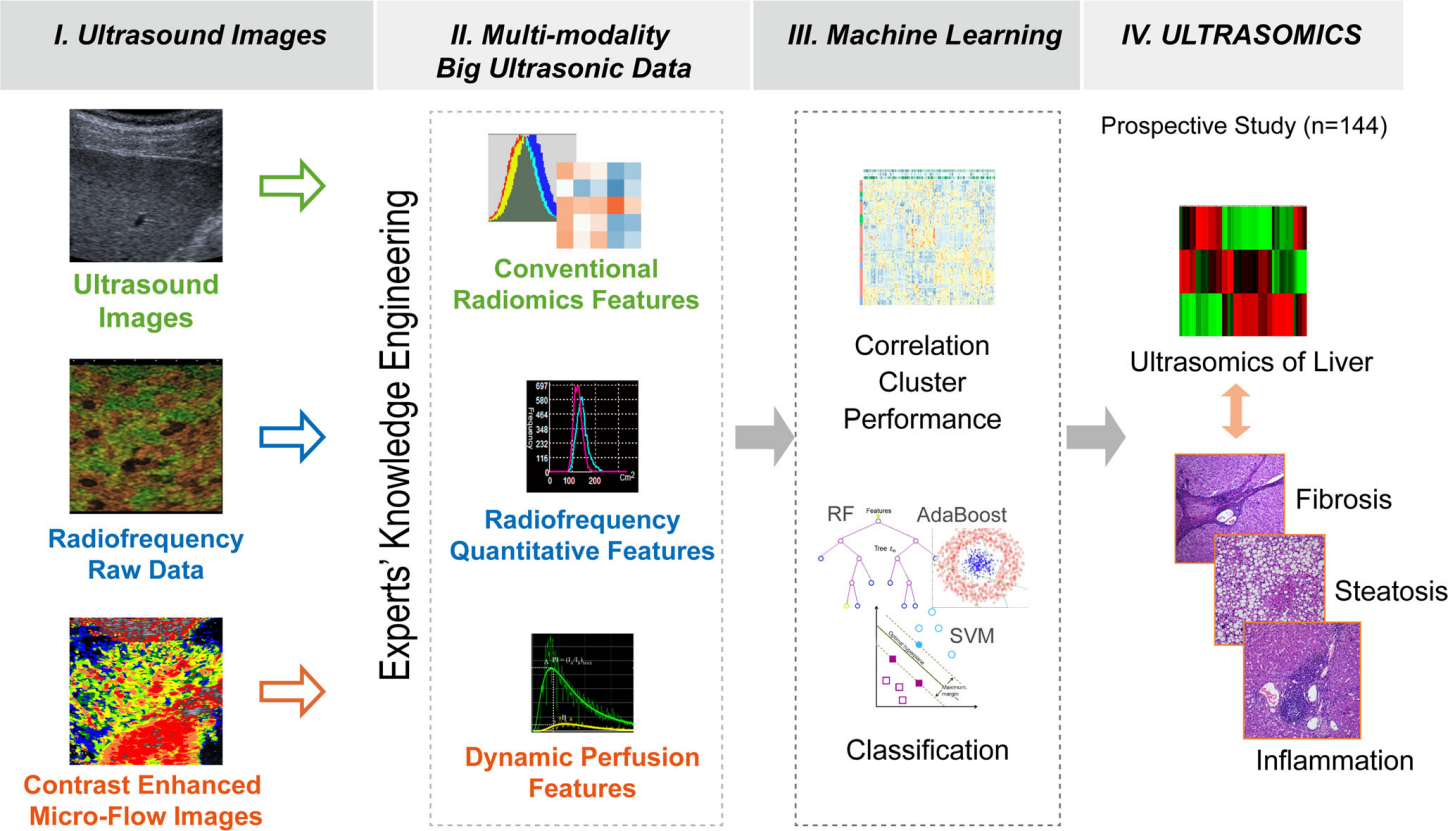
The diagram shows the four-step process for the construction of an ultrasomic-based predictive model. (I) Ultrasomic images were obtained from different US modalities (conventional images, original radiofrequency data and dynamic CEMF images). (II) Big ultrasonic data were extracted as conventional radiomic features, original radiofrequency features and dynamic CEMF features. (III) Big data mining (correlation, cluster and predictive performance) was performed to select the optimal predictor, and the classification performance of ultrasomic models was tested via machine learning. (IV) In this study, we prospectively enrolled 144 patients with liver fibrosis to establish an ultrasomic model for the prediction of fibrosis stages

According to our preliminary and reported studies [10, 11, 19–21], the three categories of parameters (conventional radiomics, ORF and CEMF features) acquired from the three types of images were expected to provide potential information for liver fibrosis staging and are detailed in the Supplementary Materials. In total, there were 472 features, including 396 conventional radiomics, 54 ORF and 22 CEMF features. These computed quantitative features were selected to construct the ultrasomics—the “omics” data of ultrasound in this study (Fig. 1).

### Multiparameter-based ultrasomic analysis of liver fibrosis using machine learning

#### Feature selection and analysis of multiparametric ultrasomics

Spearman’s correlation coefficient (R) was used to assess correlations between features in all parameters. Feature pairs with |R| > 0.90 were considered to be highly correlated and likely to provide redundant rather than complementary information. The highly correlated features were collapsed into one representative feature, usually the one with the greatest variability or highest dynamic range. This procedure yielded independent features for conventional radiomics, ORF and CEMF features.

Then, we explored feature correlations by establishing a correlation map for pairwise associations among the three categories of parameters. A hierarchical cluster of all quantitative features was plotted with different stages of fibrosis, activity and steatosis. The performance of each feature was further quantified by calculating the area under the receiver-operator characteristic curve (AUC) for fibrosis stage, activity and steatosis.

#### Multiparametric ultrasomic-based models for significant fibrosis using machine learning

Three categories of parameters with AUCs > 0.6 for assessing all stages of fibrosis were selected for the following analysis. To assess the optimal machine-learning method, all parameters of the three categories were selected for model construction. A total of six types of machine-learning algorithms—adaptive boosting (AdaBoost), decision tree (DT), logistic regression (LR), neural network (NN), random forest (RF) and support vector machine (SVM)—were tested in this study. These machine-learning algorithms were selected because of their promising performance in classification [13]. The brief descriptions of each classifier were explained in the Supplementary Material.

The entire cohort was randomly divided into a training data set (100 cases) and validation data set (44 cases). The training data set was used to compose a model and evaluated it by a validation data set. Six models with different machine-learning methods were built on the training data set, and the performance of each model was then assessed on the validation data set. To ensure the robustness of the classifiers to training and testing data, we adopted a ten-fold cross-validation method to calculate the diagnostic value for significant fibrosis (≥ F2). All processes were repeated ten times with random seed, resulting in ten different training and validation data sets. We repeatedly composed a model using a training data set and evaluated it by a validation data set, and a model that showed the best classification performance was chosen as the best model. The classification performance for significant fibrosis was assessed using the AUC in the validation data sets.

### Comparison of multiparametric ultrasomics models

Multiparametric ultrasomics models using optimized machining-learning methods were compared against models of three categories of parameters combined: (1) a combination of conventional radiomics, ORF and CEMF; two categories of parameters combined: (2) a combination of conventional radiomics and CEMF, (3) a combination of ORF and CEMF, (4) a combination of conventional radiomics and ORF; and models with a single parameter: (5) conventional radiomics, (6) ORF and (7) CEMF. Classifier performance was assessed by computing the accuracy, sensitivity, specificity and receiver-operating characteristic (ROC) curve. The AUCs for significant fibrosis in the validation data sets were assessed with the adopted ten-fold cross-validation method.

### Statistical analysis

Statistical analyses were performed with the open-source statistical computing environment R (version 3.3.1; R Foundation for Statistical Computing). We filtered features of three modalities based on independence from other features (intraclass Pearson correlation, |r| > 0.9). Heat maps of interclass Pearson correlations among the three categories of parameters were calculated and plotted using the R package “*corrplot*”. Six machine-learning algorithms were applied with the R packages “*rpart*”, “*ada*”, “*randomForest*”, “*kernlab*”, “*rms*” and “*nnet*”. AUCs for staging fibrosis, activity and steatosis were explored for the three categories of features with the R package “*pROC*”. The differences between model performance across different machine-learning and parameter subgroups were evaluated via a permutation test by using the R package “*Deducer*”. Coefficients of variation (CVs) were calculated to compare the discrete degrees of AUCs. All statistical tests were two-sided, and *p* values < 0.05 were considered statistically significant.

## Results

### Patient characteristics

A total of 144 patients were enrolled in the study, of whom 114 were male. The mean BMI was 20 kg/m^2^, and the mean age was 48 years. METAVIR fibrosis, activity and steatosis distribution are summarized in Table 1.

**Table 1:** Demographic and clinical characteristics of patients

Characteristic (*n* = 144)	Value
Age (years) ^#^	48.24 ± 13.75
Gender (male/female)	114/30
Body mass index (kg/m^2^)^#^	20.20 ± 3.16
Hepatitis B surface antigen (+/-)	98/46
Platelet count (×10^9^/l)*	5.70 (4.69-7.22)
ALT level (U/l)*	31.5 (20.5-50.0)
AST level (U/l)*	30.0 (23.0-42.5)
AST/ALT*	1.0 (0.734-1.262)
Albumin level (g/l)*	41.8 (38.6-44.45)
Total bilirubin level (μmol/l)*	12.55 (9.45-18.30)
γ-GL level (U/l)*	53.5 (32.0-122.5)
Prothrombin time (s)*	12.60 (12.10-13.40)
APRI*	0.396 (0.281-0.733)
FIB4*	1.344 (0.885-2.248)
HBV-DNA (<100/>100 cps/ml)	62/82
Fibrosis (F0/F1/F2/F3/F4)	15/33/38/23/35
Inflammation (A0/A1/A2/A3)	9/70/50/15
Steatosis (S0/S1/ S2/S3)	101/36/5/2

Note: Unless otherwise indicated, data are number of patients

*ALT* alanine aminotransferase, *AST* aspartate aminotransferase, *GL* gamma-glutamyl transpeptidase, *APRI* aspartate aminotransferase to platelet ratio index, *FIB4* fibrosis-4 index

*Data are medians, with interquartile range in parentheses

^#^Data are means ± standard deviation

### Feature selection and analysis of multiparametric ultrasomics

**Fig. 2:**
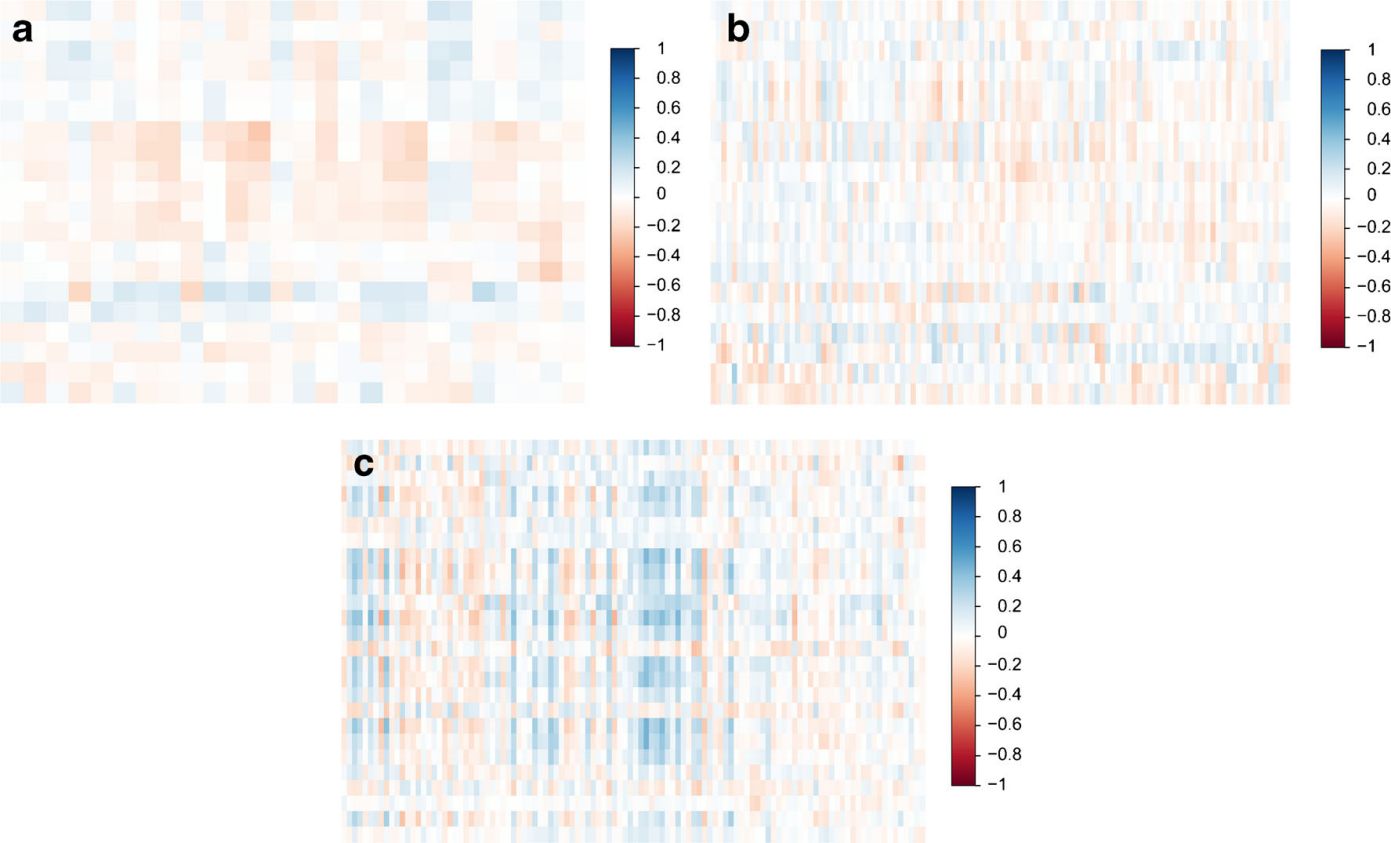
The correlation heat map shows associations between conventional radiomics, ORF and CEMF features. Only a few parameters were highly correlated (blue) or highly anti-correlated (red). In general, we found that these features were only slightly or moderately correlated between ORF and CEMF features (**a**, Spearman rho, -0.26 to 0.24) and CEMF and conventional radiomic features (**b**, Spearman rho, -0.26 to 0.32). However, the variables between ORF and conventional radiomic features were more highly correlated (**c**, Spearman rho, -0.34 to 0.47)

The independent features for conventional radiomics, ORF and CEMF were 110, 20 and 26, respectively. In general, these features have barely no correlation between ORF and CEMF (Spearman rho, -0.26 to 0.24) and between CEMF and conventional radiomics (Spearman rho, -0.26 to 0.32); however, the correlation coefficient of ORF and conventional radiomics features was slightly correlated and higher than in the above two groups (Spearman rho, -0.34 to 0.47) (Fig. 2).

**Fig. 3:**
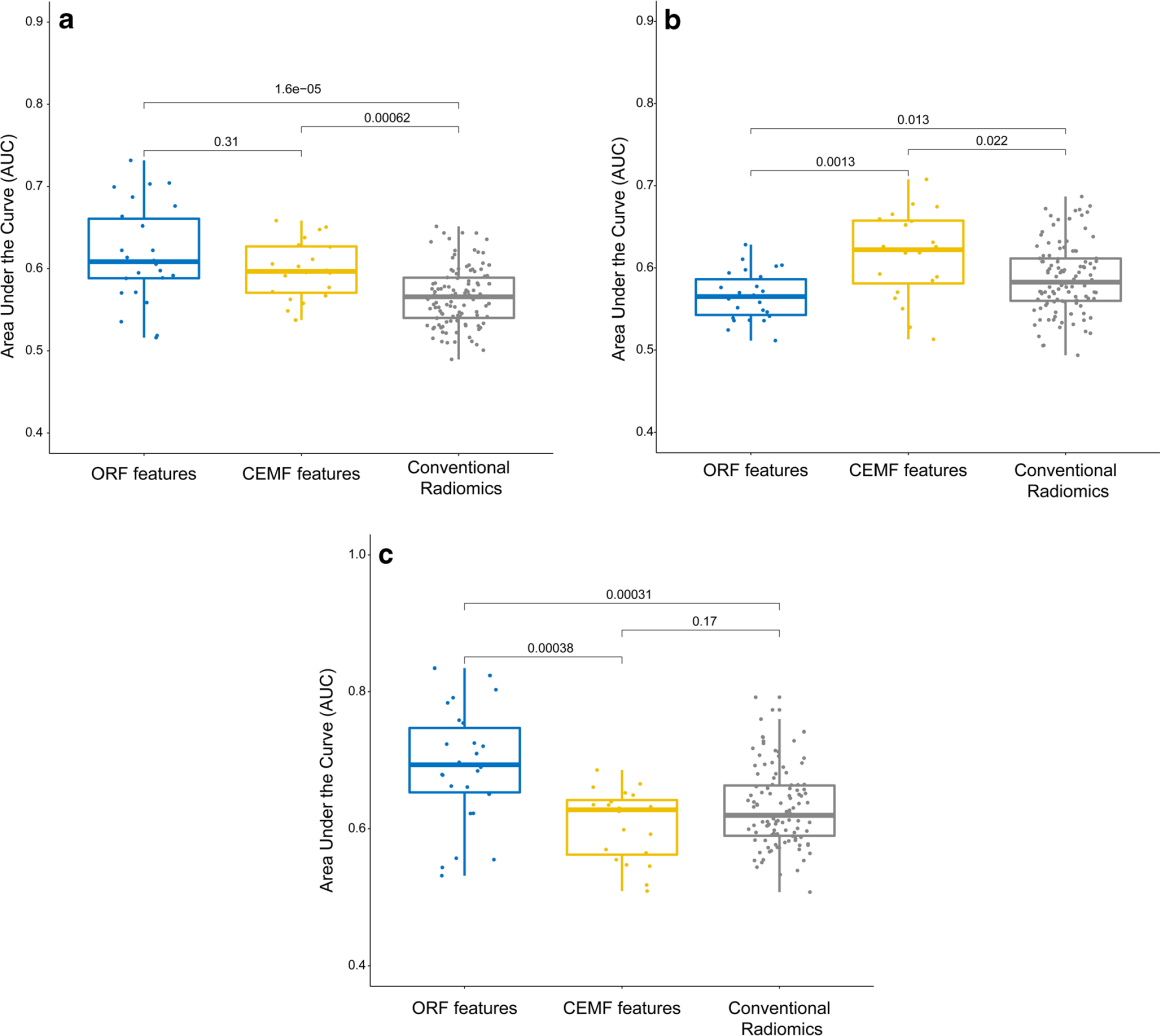
Diagnostic value of the parameters in the diagnosis of fibrosis, activity and steatosis stages. The boxplot shows that ORF and CEMF parameters, with no significant difference, were the strongest predictors and were much higher than conventional radiomic features for liver fibrosis stages (**a**, both *p* < 0.01, ANOVA test). CEMF exhibited the highest diagnostic value for activity stages (**b**, both *p* < 0.05, ANOVA test), and ORF performed the best for steatosis stages (**c**, both *p* < 0.01, ANOVA test)

Hierarchical clusters of all quantitative features were plotted with different stages of fibrosis, activity and steatosis (Fig. S1). We tested the diagnostic value of the selected parameters for fibrosis, activity and steatosis stage using AUC. A boxplot showed that ORF and CEMF features were the strongest predictors, with no significant difference, and were much stronger than conventional radiomics features for all fibrosis stages (Fig. 3a, both *p* < 0.01). Additionally, CEMF features exhibited the highest diagnostic value for all activity stages (Fig. 3b), and ORF performed the best for all steatosis stages (Fig. 3c).

### Multiparametric ultrasomic-based models for significant fibrosis using machine learning

Of the 156 features analyzed, 93 variables for conventional radiomics features, 11 variables for ORF features and 11 variables for CEMF features were removed in the final models because of their low diagnostic value (AUC < 0.6). The model for machine learning included 41 features: 17 conventional radiomics features, 15 ORF and 9 CEMF features. The detailed name and definitions of these features were listed in the Supplementary Material.

**Fig. 4:**
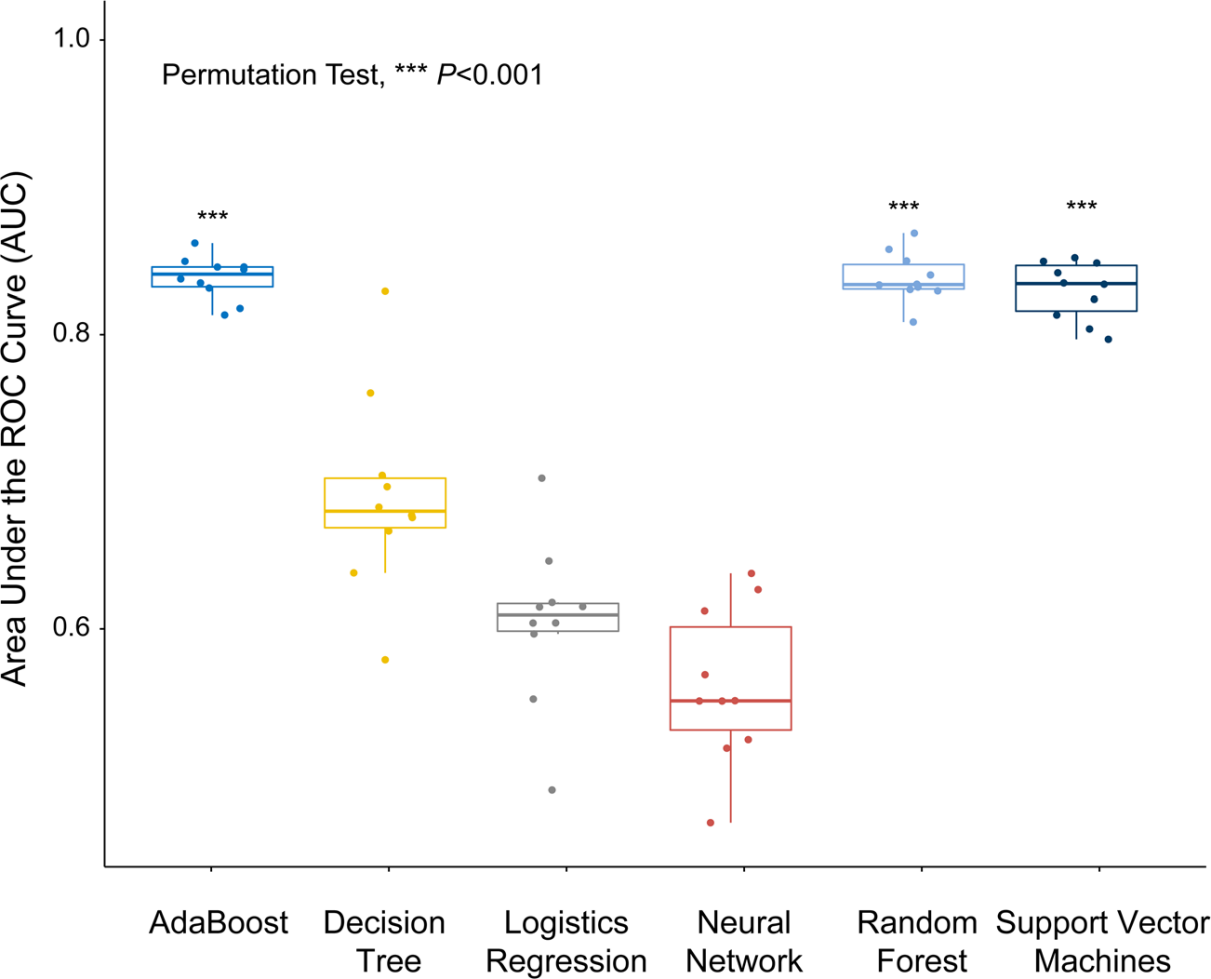
Boxplot showing the classification performance with the six machine-learning methods with all parameters. The *p* value is for a permutation test. The AdaBoost, RF and SVM classifier outperformed the other classifiers (all *p* < 0.001). These three classifiers showed less variation with a smaller quartile value and dispersion degree

The classification performance of each of the six machine-learning classification methods is shown in Fig. 4. The results showed that the AdaBoost, RF and SVM classifiers (mean AUC = 0.85 for all three) outperformed the other classifiers (mean AUC = 0.61–0.72, all *p* < 0.001). In addition, these three classifiers showed less variation, with a CV of 0.01–0.02, which was much lower than those of the other models (CV = 0.07–0.08). Based on these findings, the AdaBoost, RF and SVM classifiers were selected for the next computing because of their stability, effectiveness and high staging performance.

### Comparison of multiparametric ultrasomics models

**Fig. 5:**
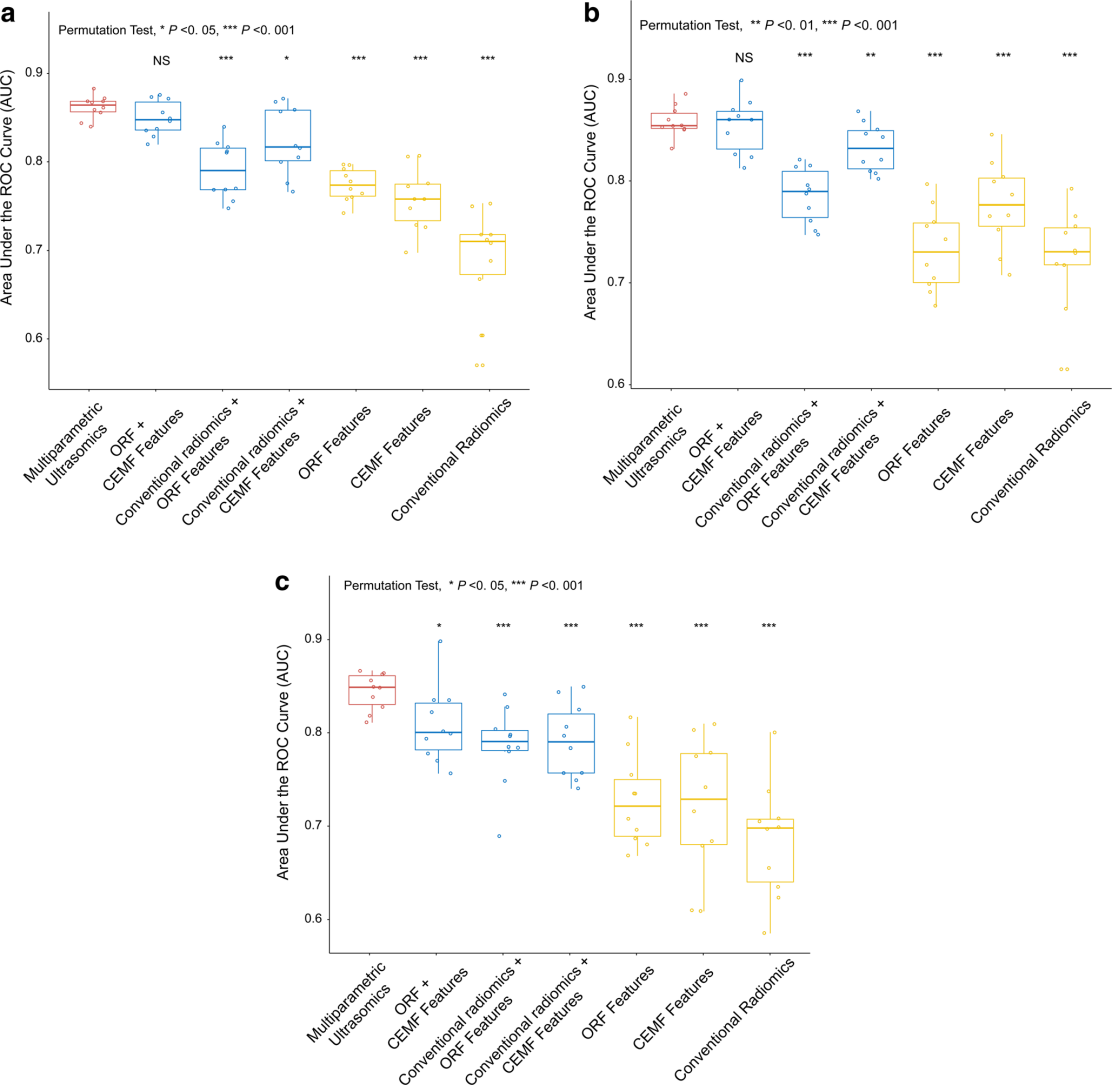
The results of three machine-learning method analyses in classifying each combination of ultrasomic features. All three machine-learning methods-adaptive boosting (**a**), random forest (**b**) and support vector machine (**c**) showed that the ultrasomics models achieved much better performance than the models of a single modality and models of conventional radiomics and ORF features (all *p* < 0.001)

The performance of machine-learning prediction models for each parameter group is summarized in Fig. 5 and Tables 2 and 3. As seen from this distribution, the combination of multiparametric features achieved much better performance (mean AUC of 0.78–0.85) than the features from a single modality (mean AUC of 0.68–0.77). For models incorporating multiparametric conventional radiomics, ORF and CEMF, classifiers of AdaBoost, RF and SVM demonstrated good performance, with the same AUC of 0.85 ± 0.01 (with 87.5%, 87.5% and 93.8% sensitivity and 76.9%, 76.9% and 69.2% specificity, respectively). For models combining two modalities, combinations of ORF and CEMF and combinations of conventional radiomics and CEMF demonstrated good performance, with mean AUCs of 0.82–0.85; however, the performance of the models combining conventional radiomics and ORF was poorer, with a mean AUC of 0.78–0.79. The models with a single modality showed only fair performance, with a mean AUC of 0.68–0.77.

**Table 2:** Training and validation results from machine learning-based classification of ultrasomics features

Features	Adaboost		Random forest		Support vector machine	
	Training	Validation	Training	Validation	Training	Validation
CR, ORF and CEMF	0.97 ± 0.02	0.85 ± 0.01	1.00	0.85 ± 0.01	0.94 ± 0.01	0.85 ± 0.01
CR and CEMF	0.97 ± 0.03	0.82 ± 0.04	1.00	0.83 ± 0.02	0.91 ± 0.02	0.80 ± 0.03
ORF and CEMF	0.97 ± 0.02	0.84 ± 0.02	1.00	0.85 ± 0.03	0.91 ± 0.02	0.82 ± 0.04
CR and ORF	0.97 ± 0.01	0.78 ± 0.03	1.00	0.78 ± 0.03	0.91 ± 0.03	0.79 ± 0.04
CR	0.95 ± 0.04	0.68 ± 0.06	1.00	0.72 ± 0.05	0.84 ± 0.04	0.71 ± 0.05
ORF	0.95 ± 0.02	0.77 ± 0.02	1.00	0.73 ± 0.04	0.90 ± 0.03	0.74 ± 0.04
CEMF	0.97 ± 0.02	0.75 ± 0.03	1.00	0.77 ± 0.04	0.91 ± 0.05	0.74 ± 0.06

Note: Performance metrics are from hold-out samples (based on ten-fold cross-validation). Data in the table are mean ± standard deviation

*CR* conventional radiomics, *ORF* original radiofrequency, *CEMF* contrast-enhanced micro-flow

**Table 3:** Performance metrics from machine learning-based classification of ultrasomic features

Features	Adaboost			Random forest			Support vector machine		
	AUC	Sensitivity (%)	Specificity (%)	AUC	Sensitivity (%)	Specificity (%)	AUC	Sensitivity (%)	Specificity (%)
CR, ORF and CEMF	0.85 ± 0.01	87.5	76.9	0.85 ± 0.01	87.5	76.9	0.85 ± 0.01	93.8	69.2
CR and CEMF	0.82 ± 0.04	59.4	100	0.83 ± 0.02	71.9	92.3	0.80 ± 0.03	81.3	76.9
ORF and CEMF	0.84 ± 0.02	92.9	71.4	0.85 ± 0.03	92.9	71.4	0.82 ± 0.04	100	71.4
CR and ORF	0.78 ± 0.03	59.4	100	0.78 ± 0.03	56.3	92.3	0.79 ± 0.04	81.3	84.6
CR	0.68 ± 0.06	43.8	100	0.72 ± 0.05	50.0	92.3	0.71 ± 0.05	90.6	46.2
ORF	0.77 ± 0.02	81.3	69.2	0.73 ± 0.04	62.5	84.6	0.74 ± 0.04	87.5	69.2
CEMF	0.75 ± 0.03	78.1	69.2	0.77 ± 0.04	84.4	76.9	0.74 ± 0.06	90.6	53.9

Note: Performance metrics are validation results from hold-out samples (based on ten-fold cross-validation). Data of AUCs in the table are mean ± standard deviation

*CR* conventional radiomics, *ORF* original radiofrequency, *CEMF* contrast-enhanced micro-flow, *AUC* area under the receiver-operating characteristic curve

## Discussion

In the current study, we propose the use of multiparametric ultrasomics as a decision support tool for liver fibrosis staging. In addition to conventional radiomics features from digital images, we acquired RF signal and dynamic perfusion information to construct ultrasomics, which are unique but convenient to acquired ultrasound parameters [10, 11, 20]. These mineable data for the evaluation of fibrosis staging were tested and compared with different machine-learning algorithms. Multiparametric ultrasomics using AdaBoost, RF and SVM provided the highest performance in this study with a small sample size.

In the construction of ultrasomics, we used unsupervised machine learning to explore the data characteristics of the parameters. A higher correlation between ORF and conventional radiomics features was found. An ORF signal is post-beam-formed data from a transducer, and it can provide intact information without signal processing [22]. Radiomics parameters are conventional features that are mathematically extracted quantitative descriptors based on digitally transformed images [23]. Although the digital images were signals post-processed with a digital scan converter, both signals simulated the morphological homogeneity of liver tissue. Notably, the ORF data, which included original and superior information, performed better in the assessment of fibrosis and steatosis. Some papers have reported results on the tissue characterization of hepatic fibrosis or steatosis via quantitative ultrasound examination using statistical data on B-mode ultrasound and radiofrequency echo signals [24–26]. However, dynamic perfusion parameters, showing a lower correlation with both morphological parameters, demonstrated the highest diagnostic value for activity stages, which was correlated with liver microcirculation [27]. Therefore, our results suggested that these three signals could be divided into two categories: morphology and hemodynamics, which reflected the fibrosis and steatosis stages and the activity stages, respectively.

For liver fibrosis staging with ultrasomics, although the optimized machine-learning algorithms had been selected, a clinical model that used single-modality parameters still provided unsatisfactory AUC values for staging (AUC < 0.8). In addition, the models that used duplicate morphological parameters (higher correlated features of ORF and conventional radiomics) displayed the lowest AUCs in the validation groups, which may be due to the redundant information between two morphological parameters. Moreover, the models using combined morphology and hemodynamic features demonstrated better performance. For the evaluation of fibrosis stage, the accompanying activity of liver tissue should not be ignored [28, 29]. Our results also agree with the principle that models constructed with two categories should achieve higher AUCs despite the use of a machine-learning algorithm. This finding shows that the use of multiparametric ultrasomics from different pathophysiological procedures would enhance the performance of our clinical decision support system [30].

With these big data, machine learning is driving great changes in medical disciplines that are based on pattern recognition (e.g., radiology and pathology) [31, 32]. Generally, machine learning with a larger sample size produces more accurate classification [32–34]. However, the ultrasound images that qualified for the computing analysis in the present work were restricted because of their dependence on the operator and software, and our study included only 144 cases as a result. Therefore, determining the optimal machine-learning algorithm for a small sample size is of great value. A few recent studies have investigated the effects of different machine-learning classification methods on single-modality radiomic-based clinical predictions [35, 36]. Our study showed that the three machine-learning methods of AdaBoost, RF and SVM performed better with any category of parameters. The principle of SVM is to map the input parameters into a high-dimensional feature space via preselected nonlinear mapping [33, 37]. In this space, an optimal classification hyperplane is constructed and is optimized to maximize the classification of the two categories. The use of a margin among the hyperplane and two categories reduces the size and distribution requirements of the data. RF combines predictions from several weak classifiers to generate a more accurate and stable prediction. Random samples and features guarantee the robustness to noise in the data with few tuning parameters and a small sample size [32, 38, 39]. AdaBoost is adaptive in the sense that subsequent weak learners are tweaked in favor of instances misclassified by previous classifiers [13, 36]. Thus, AdaBoost is sensitive to noisy data and outliers; this sensitivity makes AdaBoost less susceptible to the overfitting problem.

The first and most important limitation of this study was that multiparametric ultrasomics could not include parameters of liver stiffness. Many single-center studies have already shown that shear wave elastography achieved a good AUC of 0.89 in staging F2 fibrosis patients [17]. However, considering patient compliance, only one US machine was used to acquire the ultrasound data in this prospective study. Additionally, the primary purpose of this study was to optimize the model of fibrosis staging based on multiparametric ultrasomics using machine learning. Our present work has demonstrated that (1) data exploration correlating with pathophysiology and (2) model construction using machine learning could improve the robustness of fibrosis staging models. However, the reported performance of shear wave elastography in staging significant fibrosis (AUC = 0.69–0.92, sensitivity = 0.77–0.90 and specificity = 0.70–0.87) was similar to that of our multiparametric model. This finding reminds us that, although the data mining process could enhance the performance of clinical decision systems, the optimal performance of the model has already been determined by the data. In a subsequent study, we will collect shear wave elastography data to produce a better model.

Our study also had other limitations. Second, our study only included a cohort of patients in our hospital. It is necessary to establish an independent validation cohort to test the generalizability of our ultrasomics model. A third limitation is that several patients enrolled in the study had focal liver lesions. This may also affect the association between US parameters and pathology. To eliminate the potential effect of tumors on the adjacent liver parenchyma, we created strict exclusion criteria. Fourth, the population of the study was Chinese patients with chronic HBV, which led to a low BMI of 20.2 kg/m^2^ and a low proportion of liver steatosis. Fifth, due to the requirement in image acquisition, only a short time (15–20-s clips) was covered in the CEMF images. This may not show the whole perfusion procedure in the liver. However, we attempted to analyze the blood flow arrival time to the liver and kidney based on a time-intensity curve, and the wash-in curve contains more information for the arrival time. Sixth, the analysis of the liver (conventional radiomics and ORF) was done from a small 2-cm ROI placed in segment 6, which has the same limitation as a biopsy of not reflecting the potential heterogeneity of the liver disease.

In summary, we have demonstrated that expert knowledge on data acquisition and analysis can optimize the robustness of clinical decision support systems. Additionally, the three machine-learning methods of AdaBoost, RF and SVM are optimal algorithms for studies with a small sample size. The application of this framework in future studies will facilitate data mining in the era of ultrasomics.

## Electronic supplementary material

ESM 1 (DOCX 3527 kb)

## Abbreviations

AdaBoost: Adaptive boosting
CEMF: Contrast enhanced micro-flow
CV: Coefficient of variation
DT: Decision tree
LR: Logistic regression
NN: Neural network
ORF: Original radiofrequency
RF: Random forest
SVM: Support vector machine
