# Supplementary material for: Multiparametric ultrasomics of significant liver fibrosis: A machine learning-based analysis
Source: Eur Radiol. 2018 Sep 3;29(3):1496–506. doi: 10.1007/s00330-018-5680-z (PMC6510867; doi:10.1007/s00330-018-5680-z)
Supplement: Supplementary file 1 — (DOCX 3527 kb) [file 330_2018_5680_MOESM1_ESM.docx]

**Supplementary Materials**

1. Inclusion and exclusion criteria of the study

2. Patient characteristics

3. Feature extraction of multi-modality ultrasomics

3.1 Conventional radiomics

3.1.1 Statistics features

3.1.2 Textural features

3.1.3 Wavelet features

3.2 Original signal features

3.3 Dynamic CEMF features

4. Explanations of the six classifications

4.1 AdaBoost

4.2 Decision tree (DT)

4.3 Logistic regression (LR)

4.4 Neural network (NN)

4.5 Random forest (RF)

4.6 Support vector machine (SVM)

5. Selected features in the model

6. Supplementary Figures

7. Supplementary material reference

**1. Inclusion and exclusion criteria of the study**

The following inclusion criteria were used: (1) patients were confirmed to have chronic hepatitis B virus (HBV) infection; HBV infection was characterized by serology test results that were positive for HBV surface antigen and/or HBV DNA; None of the patient were receiving antiviral therapy at the time of enrollment. (2) availability of all three modality ultrasound (US) examinations were performed within the 2 weeks before liver biopsy or partial liver resection; (3) adequate liver specimens were assessed by pathological examination (more than six portal tracts under the microscope).

The exclusion criteria were as follows: (1) patients with a maximum tumor diameter of 5 cm or larger (n = 137); a tumor located in segment 6 of the liver, as the acquisition section for US images in the study was on segment 6 (n = 53); (2) intrahepatic cholangiectasis caused by hilar cholangiocarcinoma or tumor compression (n = 16); (3) inflammation in other organs or circulation system disease (n = 12); (4) abnormal renal function (n = 8).

**2. Patient characteristics**

Basic clinical data, including age, gender, body weight, height, and body mass index, were recorded on the same day as the US examination after the patients had fasted overnight (8 hours). Laboratory tests included (1) serology markers, including alanine aminotransferase (ALT), aspartate aminotransferase (AST), γ-glutamyltransferase (GGT), alkaline phosphatase (ALP), total bilirubin (TBIL), serum albumin (ALB), prothrombin activity (PT), platelet count (PLT), and HBV DNA.

Liver fibrosis indexes, including the aminotransferase to platelet ratio index (APRI) and fibrosis-4 index (FIB-4), were previously demonstrated to be efficient in the estimation of liver fibrosis and were used in this study. The serum algorithm models were calculated as follows: APRI = AST/UL×100/PLT, where the upper limit (UL) was considered to be 40 U/L (1); $FIB-4=(A\times AST)/(\sqrt{ALT}\times PLT)$, where A is age (in years), AST and ALT were measured in units per liter, and PLT was measured in 10^9^ per liter (2).

**3. Feature extraction of multi-modality ultrasomics**

**3.1 Conventional radiomics**

Conventional image data were analyzed (took up 230-550KB for one case). A circular region of interest (ROI) with a diameter of 2.0 cm was manually delineated in segment six of the liver, and intrahepatic visible vessels and bile ducts were avoided. All feature extraction methods were implemented with A.K. software (Artificial Intelligence Kit, version 1.1, GE Healthcare). A total of 396 features were extracted and included three types: statistics features, textural features, and wavelet features; these features are described below.

**3.1.1 Statistics features**

Statistics features describe the distribution of grayscale voxel intensities within the conventional image through commonly used and basic metrics. To analyze the distribution of the pixels’ hue matrix and extract static features of images, a fuzzy similitude matrix is defined. The matrix describes the image’s feature distribution. The following statistics features were extracted: energy, entropy, kurtosis, maximum, mean, mean absolute deviation, median, minimum, range, root mean square (RMS), skewness, standard deviation, uniformity, and variance.

**3.1.2 Textural features**

Textural features can quantify intratumor heterogeneity in the texture and the arrangement of properties that change slowly or periodically within the tumor. The features were calculated from three matrices: the gray level co-occurrence matrix (GLCM), the gray level run-length texture matrix (GLRLM), and the gray level size zone matrix (GLSZM).

The GLCM is the matrix function that describes the distance and angle of each pixel and can reflect integrated information regarding the direction, interval, amplitude, and frequency of images. The textural features in the GLCM mainly consisted of autocorrelation, cluster prominence, cluster shade, cluster tendency, contrast, correlation, difference entropy, dissimilarity, energy, entropy, homogeneity, inverse difference moment normalized, inverse difference normalized, inverse variance, maximum probability, sum average, sum entropy, sum variance, and variance.

The GLRLM quantifies gray level runs in an image. The gray level run is the length of consecutive voxels having the same intensity in a preset direction in the image, whereas the zone is a cluster of consecutive voxels having the same intensity. A GLRLM is a two-dimensional matrix in which each element p (i, j|α) gives the total number of occurrences of runs of length j at gray level i in a specific direction α. The features in the GLRLM mainly consist of short run emphasis (SRE), long run emphasis (LRE), gray level non-uniformity (GLN), run-length non-uniformity (RLN), run percentage (RP), low gray level run emphasis (LGLRE), high gray level run emphasis (HGLRE), short run low gray level emphasis (SRLGLE), short run high gray level emphasis (SRHGLE), long run low gray level emphasis (LRLGLE), and long run high gray level emphasis (LRHGLE).

The GLSZM quantifies size zone matrices in an image. By defining the connected voxels with the same gray level as a zone, a matrix with 2^3^ rows is then deduced, in which the element at row r and column s stores the number of the zone with gray level r and size s. The number of columns in this matrix is determined by the size of the largest zone. Therefore, a wide and flat matrix indicates that the texture information is homogeneous in the predefined ROI, while a narrow matrix indicates heterogeneity. The features in the GLSZM mainly consist of small area emphasis (SAE), large area emphasis (LRE), intensity variability (IV), size zone variability (SZV), zone percentage (ZP), low-intensity emphasis (LIE), high-intensity emphasis (HIE), low-intensity small area emphasis (LISAE), high-intensity small area emphasis (HISAE), low-intensity large area emphasis (LILAE), and high-intensity large area emphasis (HILAE).

**3.1.3 Wavelet features**

The wavelet transform was used to decompose the original image and can be regarded as a preprocessing step prior to feature extraction. The wavelet transform increases the information of the low-frequency signal by changing the ratio of high-frequency to low-frequency signals. The signals were decomposed into decomposing images. The size of each decomposition is equal to the original image. For each decomposition, we computed the statistics and textural features described above.

**3.2 Original signal features**

ORF parameters (took up 15-20MB for one case) were automatically extracted using our built-in algorithm through off-line analysis, which was modified from acoustic structure quantification (ASQ, Version 1.11R001) parameters. Three ROIs with a diameter of 2.0 cm were drawn manually on one image, avoiding large hepatic vessels or bile ducts. ORF is based on the statistical chi-square test of acquired radiofrequency signals of raw data (3). It compares the difference between theoretical and real signal amplitude distribution. Theoretical echo amplitude is thought to be a function of the Rayleigh distribution based on the assumption that the speckle pattern is induced only by US beam interference of very small scattering objects, which are located closer than the US wavelength. However, real echo amplitude of the liver parenchyma does not fit a Rayleigh distribution, mainly due to the presence of small structures, such as hepatic vessel walls, that scatter the US beam. Besides, fibrotic structures, including cirrhotic nodules and blood vessel walls, increase in liver fibrosis. Consequently, the echo signals from a fibrotic liver tend to be more unlikely to be a theoretical echo amplitude and inconsistent with the Rayleigh distribution (3, 4). In ORF examination, once a primary ROI (pROI) is set manually on the image, hundreds of secondary ROI (sROI) are automatically set sweeping pROI to complete the analysis. The basic analysis parameter, defined as C^2^m, measures the difference between the theoretical and observed distributions of echo amplitude, and can be calculated by the following equation.


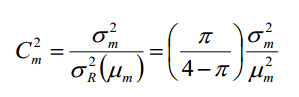


whereμ_m_ and σ_m_ are the average and variance, respectively, of the echo amplitude in a sROI.

The results for C^2^m were plotted on a histogram. The C^2^_m_^’^ was recalculated from the limited samples less thanμ_m_ +4σ_m_. When the ratio C^2^_m_ /C^2^_m_^’^ is larger than threshold α, the result of C^2^_m_ is eliminated from the histogram (full line), but added to the alternative histogram (dotted line) (Fig. S2). Then, the peak, average, and standard deviation (SD) of the C^2^_m_ values for total, full line, and dotted line are expressed as total_peak, total_average, total_SD, full_peak, full_average, full_SD, dotted_peak, dotted_average, and dotted_SD, respectively. Then, the AUC ratio is calculated as the ratio of the area under the full line and dotted line:

AUC ratio = [AUC (pink solid)/AUC (cyan dotted)]

A total of 54 variables were extracted from the ORF images, including the conventional parameters of ASQ. The 144 cases were collected between October 2013 and April 2015, which overlapped our previous study with 36 cases. We explored the computing quantitative parameters up to 54 variables using our built-in algorithm.

**3.3 Dynamic CEMF features**

One ROI was drawn in the parenchyma of the right kidney, and the long axis of the ROI should be parallel to the long axis of the renal parenchyma. Three ROIs with diameters of 2.0 cm were set in the liver, avoiding large hepatic vessels or bile ducts, and set at 1-2 cm deeper than the liver capsule of the liver parenchyma to avoid the variation from tissue attenuation. Dynamic CEMF features (took up 3-7MB for one case) were extracted via our built-in algorithm through off-line analysis.

The detailed principle of a dynamic CEMF image was based on the understanding that the liver received dual blood supply from the hepatic artery and portal vein. The hepatic arterial phase was supplied by two flow in pathways (the hepatic artery and portal vein flow into the liver with the constant of K_1a_ and K_1p_, respectively) and one flow out pathway (the hepatic vein flows out of the liver with the constant of K_2_).

$$C_{L}\left( t \right)=\int_{0}^{t} [K_{1a}C_{a}\left( t^{'}-ta \right)+K_{1p}\mathrm{Cp}\left( t^{'}-t_{p} \right)]e^{k2(t^{'}-t)}dt'$$

Ca(t), Cp(t) and CL(t) represent the density of the contrast agent at different times in the hepatic artery, portal vein and liver parenchyma, respectively. ta and tp represent the arrival time of the contrast agent in the hepatic artery and portal vein, respectively.

The kidney was regarded as purely arterial blood supply. The dual blood supply of the liver was compared to the solely arterial blood supply to obtain the blood supply from the portal vein. Three categories of parameters were analyzed: a. time parameters: starting time (ST), mean time (MT), enhancement time (ET) and adjusted arrival time (AAT) for comparison between liver and kidney; b. area parameters: area of artery blood supply (AA), area of venous supply (AV), and ratio of AA and AV (RAV); and c. slope parameters: slope and fitting slope of the kidney and liver (S_K_ and S_L_), ratio of S_K_ and S_L_.

**4. Explanations of the six classifications**

**4.1 AdaBoost**

AdaBoost is an abbreviation for adaptive boosting. It is a meta-learning algorithm that combines result of various "weak" classifiers namely Least Square (LS), Normal Density Discriminant Function (NDDF), Perceptron, Pocket and Stumps adaptively to improve the classification performance. The final model is then an additive model constructed from the sequence of models. Boosting increases the weights of the less accurate algorithms. Sequences of models are modified to give more weight to those observations that are more difficult to classify.

**4.2 Decision tree (DT)**

DT is a traditional computer science structure for organizing data. DT can represent both classification and regression models. It constructs a tree from the training data by using the selected features. The process starts with a single root node that splits into multiple branches, leading to further nodes, each of which may further split or terminate as a leaf node that is not further split and contains the “decisions.” The structure can represent both classification and regression models. DT is easy to view, understand, and explain. It does not always deliver the best performance but represents a trade-off between performance and the simplicity of explanation.

**4.3 Logistic regression (LR)**

Logistic regression measures the relationship between the categorical dependent variable and one or more independent variables by estimating probabilities using a logistic function, namely, the cumulative logistic distribution. This approach is used to estimate the probability of a binary response based on one or more predictor (or independent) variables (features).

**4.4** **Artificial Neural network (ANN)**

ANN is an information processing paradigm that is inspired by the way biological nervous systems, such as the brain process information.. It is composed of a large number of highly interconnected processing elements (neurones) working in unison to solve specific problems. It is an adaptive system that changes its structure based on external or internal information that flows through the network. Such systems learn (progressively improve performance) to perform tasks by considering examples, generally without task-specific programming. The connections of the biological neuron are modeled as weights. A positive weight reflects an excitatory connection, while negative values mean inhibitory connections. All inputs are modified by a weight and summed. This activity is referred as a linear combination. Finally, an activation function controls the amplitude of the output. To select and tune an algorithm, the correct hyperparameters for training on a particular data set are needed and considerable experimentation is required.

**4.5** **Random forest (RF)**

The RF algorithm describes an approach to building models where the actual model builder could be a DT algorithm, a regression algorithm, or any one of many other model building algorithms. RF is commonly presented in terms of DTs as the primary form for the representation of knowledge. RF can be thought of as a meta-algorithm. This randomness delivers considerable robustness to noise, outliers, and overfitting when compared with a single-tree classifier. RF also performs well when there are many input variables and few observations.

**4.6 Support vector machine (SVM)**

SVMs are supervised learning models with associated learning algorithms that analyze data used for classification and regression analysis. It constructs an (N-1)-dimensional hyperplane that classifies the data into two categories where N represents the number of input features. The hyperplane acts as a decision surface separating two classes with a maximum margin. Both steps of feature selection were encapsulated with the classifier within each training fold in order to avoid feature selection bias and overfitting. The generated SVM model maps the features of a test data on the same space as that of training data features and predicts the class of a test data. SVMs have been found to perform well on problems that are nonlinear, sparse, and highly dimensional. The modeling only addresses support vectors rather than the whole training dataset, and thus the size of the training set is not usually an issue. In addition, the model is less affected by outliers.

**5. Selected features in the model**

5.1 Conventional radiomics features:

| Statistics features | Textural features | |
| --- | --- | --- |
|  | long run low gray level emphasis (LRLGLE) | the gray level co-occurrence matrix (GLCM) |
| standard deviation skewness histogramEntropy sumEntropy | LRLGLE_AD_O1_SD | GLCMEnergy_AD_O1 GLCMEnergy_AD_O1_SD GLCMEnergy_An0_O1 GLCMEntropy_An45_O1 ClusterShade_An135_O1 ClusterShade_AD_O4_SD GLCMEnergy_An0_O4 ClusterShade_An90_O4 ClusterShade_AD_O7_SD ClusterShade_An0_O7 ClusterShade_An45_O7 ClusterShade_An90_O7 |

5.2 Original signal features

| Full line | Dotted line | Total Mode |
| --- | --- | --- |
| SD  SD_max  SD_min  SD_max/ SD_min  Min Average.w.o.max.min | Median  max  Average_median Average_max SD_average  SD_min | SD  FD ratio_max  FD ratio_Avrage_w.o_max.min |

5.3 Dynamic CEMF features

| time parameters | area parameters | slope parameters |
| --- | --- | --- |
| Time.Hepatic_average Time.hepatic_5  Time.hepatic_50 Time.hepatic_90 Time.hepatic_95 Time.hepatic_end Time.hepatic_End.Modulation | Area_Hepatic.Artery | Slope_Renal |

**6. Supplementary Figures**

**
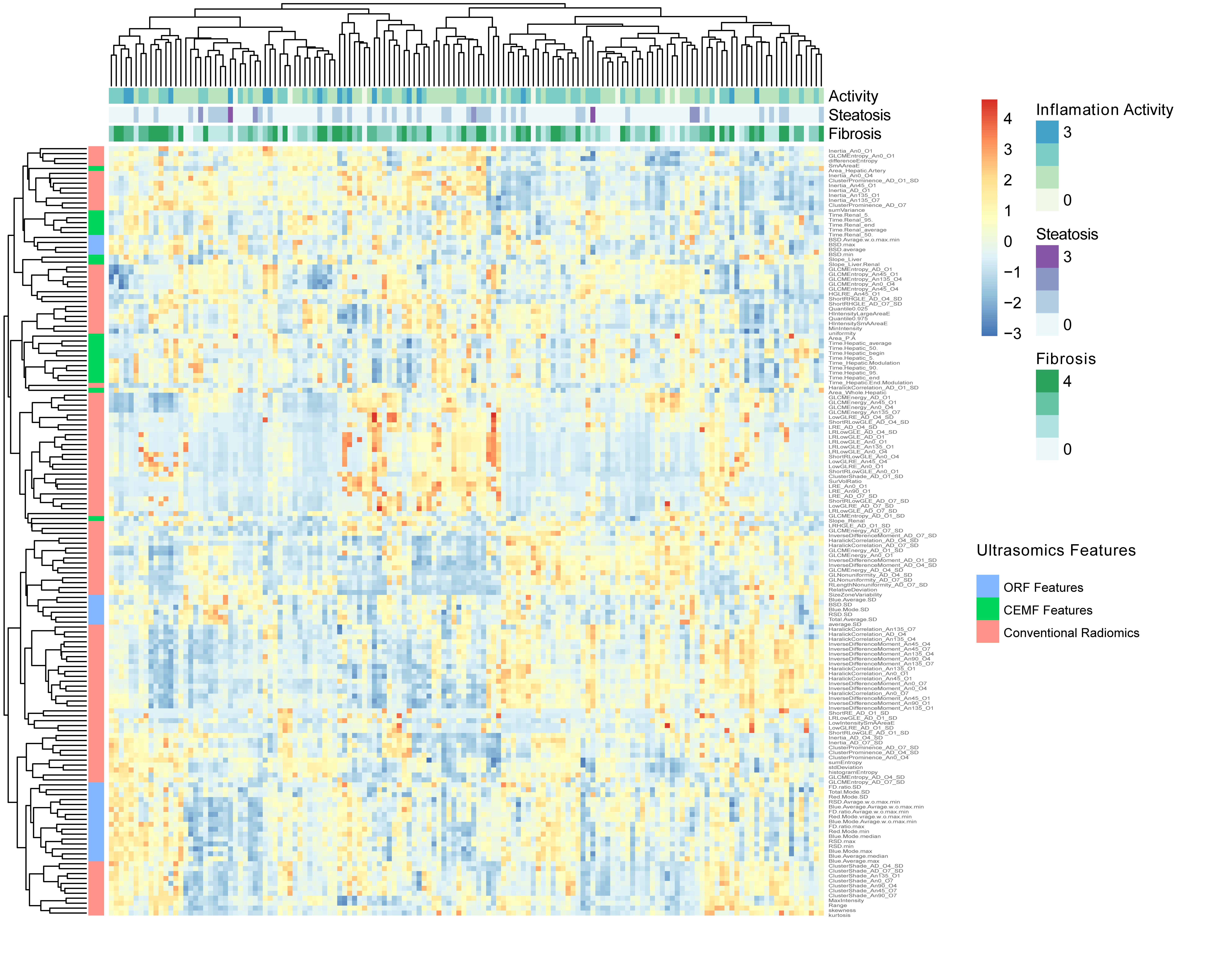
Figure S1:**

Hierarchical clustering of ultrasomics features with liver fibrosis, activity and steatosis stages. Heat map depicting Z-scores of 472 ultrasomics features for 144 patients, with cluster trees obtained from hierarchical clustering. The rows are ultrasomics features and the columns are three sub-groups of fibrosis, activity and steatosis stages.


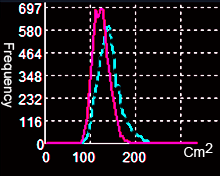


**Figure S2:**

Original radiofrequency image analysis. The difference between the theoretical (dotted line) and observed distributions (full line) of echo amplitude is presented as an occurrence histogram.

**7. Supplementary material reference**

1. Lebensztejn DM, Skiba E, Sobaniec-Lotowska M, Kaczmarski M (2005) A simple noninvasive index (APRI) predicts advanced liver fibrosis in children with chronic hepatitis B. Hepatology 41:1434-1445

2. Vallet-Pichard A, Mallet V, Nalpas B et al (2007) FIB-4: an inexpensive and accurate marker of fibrosis in HCV infection. comparison with liver biopsy and fibrotest. Hepatology 46:32-46

3. Toyoda H, Kumada T, Kamiyama N et al (2009) B-mode ultrasound with algorithm based on statistical analysis of signals: evaluation of liver fibrosis in patients with chronic hepatitis C. AJR Am J Roentgenol 193:1037-1043

4. Tuthill TA, Sperry RH, Parker KJ (1988) Deviations from Rayleigh statistics in ultrasonic speckle. Ultrasonic imaging 10:81-89
